# Nuclear factor-kappa B influences early phase of compensatory lung growth after pneumonectomy in mice

**DOI:** 10.1186/s12929-017-0350-z

**Published:** 2017-07-05

**Authors:** Yusuke Takahashi, Noriyuki Matsutani, Hitoshi Dejima, Takashi Nakayama, Hirofumi Uehara, Masafumi Kawamura

**Affiliations:** Department of General Thoracic Surgery, Teikyo University School of Medicine, 2-11-1 Kaga, Itabashi, Tokyo, 173-8606 Japan

**Keywords:** Transcription factor, SN50, Lung regeneration, Translatable model, Type 2 alveolar epithelial cells

## Abstract

**Background:**

Compensatory lung growth (CLG) is a well-established lung regeneration model. However, the sequential mechanisms, including unknown molecular triggers or regulators, remain unclear. Nuclear factor- kappa B (NF-κB) is known to be essential for inflammation and tissue regeneration; therefore, we investigated the role of NF-κB in CLG.

**Methods:**

C57BL/6 J mice underwent either a left pneumonectomy or a thoracotomy (*n* = 77). Gene microarray analysis was performed to detect genes that were upregulated at 12 h after pneumonectomy. NF-κB protein expression was examined by immunohistochemistry and Western blot. To investigate the influence of NF-κB on CLG, either an NF-κB inhibitor SN50 or saline was administered following pneumonectomy and the degree of CLG was evaluated in each group by measuring the lung dry weight index (LDWI) and the mean linear intercept.

**Results:**

Gene microarray analysis identified 11 genes that were significantly but transiently increased at 12 h after pneumonectomy. Among the 11 genes, NF-κB was selected based on its reported functions. Western blot analysis showed that NF-κB protein expression after pneumonectomy was significantly higher at 12 h compared to 48 h. Additionally, NF-κB protein expression at 12 h after pneumonectomy was significantly higher than at both 12 and 48 h after thoracotomy (*p* < 0.029 for all). NF-κB protein expression, evaluated through immunohistochemistry, was expressed mainly in type 2 alveolar epithelial cells and was significant increased 12 h after pneumonectomy compared to 48 h after pneumonectomy and both 12 and 48 h after thoracotomy (*p* < 0.001 for all). SN50 administration following pneumonectomy induced a significant decrease in NF-κB expression (*p* = 0.004) and LDWI compared to the vehicle administration (*p* = 0.009).

**Conclusions:**

This is the first report demonstrating that NF-κB signaling may play a key role in CLG. Given its pathway is crucial in tissue regeneration of various organs, NF-κB may shed light on identification of molecular triggers or clinically usable key regulators of CLG.

## Background

Compensatory lung growth (CLG) following pneumonectomy is a well-established lung regeneration model which may translate into the clinic. Recent clinical data suggests that the adult human lung may have potential of CLG to some extent [1, 2]. In our previous study, alveolar duct dilatation in the remnant right lung is the most significant immediately after left pneumonectomy. Then, the dilated alveolar duct is separated by proliferating alveolar septal cells beginning 24 h post pneumonectomy [3]. This resulted in increased number of alveoli in the remnant right lung [3] and these morphological changes resemble alveolar “septation” in normal lung development. Upon molecular analysis, upregulation of thyroid transcription factor 1 (TTF-1) was observed 12 h post pneumonectomy and TTF-1 may act as a key regulator of CLG [3, 4]. Another report recently demonstrated that angiocrine-related factors and chemokines significantly contributed to CLG [5]. Despite these recent progresses, sequential mechanisms underlying CLG remain unclear, particularly, the molecular triggers and important mediators in the CLG process.

Gene expression profiling has been utilized in various research fields including physiology and oncology in recent years [6]. It was also conducted in several studies to identify significant changes in gene expressions during lung injury [7] as well as postpneumonectomy in mice [8, 9]. Here, this array identified nuclear factor-kappa B (NF-κB) gene expression to be transiently but significantly up-regulated 12 h post pneumonectomy compared to either 48 h post pneumonectomy or post thoracotomy at 12 or 48 h.

NF-κB is an important transcription factor, which is involved in the regulation of the inflammatory response [10, 11] as well as cancer growth [12, 13]. Recent reports demonstrated that NF-κB is also involved in tissue regeneration [14, 15]. Particularly, NF-κB plays a crucial role in liver regeneration after partial hepatectomy [16, 17]. Important roles of several angiocrine-related factors in both lung and liver regeneration have been documented [5, 18]. In the current study, we aimed to investigate a role of NF-κB in CLG following left pneumonectomy in mice based on global gene expression profiling.

## Methods

### Mice preparation

Specific pathogen-free, 9-week-old, inbred, female C57BL/6 J mice (*n* = 77), weighing 20 to 22 g, were purchased from CLEA Japan, Inc. (Tokyo, Japan) and were fed a standard diet and water. The mice were maintained in a 12-h light/12-h dark cycle. All mice were treated in compliance with Guiding Principles in the Care and Use of Animals adopted by the American Physiological Society. All experiments were conducted in accordance with protocols approved by the Animal Care and Use Committee of Teikyo University. The mice were randomly assigned to the following four experimental groups: 1) thoracotomy under mechanical ventilation (THX group), 2) left pneumonectomy under mechanical ventilation (PNX group), 3) left pneumonectomy followed by saline administration (PNX + saline group), and 4) left pneumonectomy followed by SN50 administration (PNX + SN50 group).

### Surgical procedure

The following surgical procedure was performed as previously described [3, 4]. General anesthesia was induced with an intramuscular injection of ketamine (100 mg/kg) and xylazine (10 mg/kg) and then intubation was performed with 18-gauge catheter connected to a rodent ventilator, adjusting to maintain a respiratory rate of 100 breath/min, 10 mL/kg tidal volume, 2 cm H_2_O positive endo-expiratory pressure, and 0.21 FiO_2_. Then, a 30-mm-long posterolateral skin incision and thoracotomy via fifth intercostal space was made. In the THX group, the chest cavity was closed without any intrathoracic dissection at 5 min after thoracotomy. In the PNX group, the left lung was resected after *en bloc* ligation of hilus with 3–0 silk. The resection of the left lung took approximately 5 min. Then, the fifth intercostal space was closed with a single surgical suture followed by skin and muscle closure with two sutures. The duration of mechanical ventilation for the entire procedure was fixed at 10 min. The mice were returned into standard care after extubation and recovery. Then, the mice were sacrificed immediately after anesthesia, at 12 h, at 48 h, and 7 days after surgery in accordance with our previous report [3].

### RNA isolation and microarray analysis

Specifically in this gene microarray analysis, we created a negative control group of mice that did not undergo any procedure. The right lung was harvested and the vasculature was perfused with 5 mL of ice cold normal saline to flush out the blood. Equal amounts of the lung tissue of right superior lobe, excluding trachea and bronchus, were pooled from 3 mice in each group to minimize biological variability as previously described [19]. The lung was cut into small pieces and RNA Stabilization Reagent (Qiagen, Maryland, MD, USA) was added. Next, QIAzol Lysis Reagent (Qiagen) was added and the lung was homogenized on ice. Then, total RNA was extracted from the dissected lung using Qiagen RNAeasy mini kit (Qiagen) according to the manufacturer’s instruction. The RNA quality was measured using Bioanalyzer 2100 (Agilent, Santa Clara, CA, USA) and stored at −80 degrees Celsius until use. The RNA samples with an RNA concentration higher than 50 ng/μL and A260/A280 of 1.8–2.1 were used for the following microarray analysis: 250 ng of total RNA was converted to cDNA, and after amplification and Cy-3 labeling with the Low Input Quick Amp Labeling Kit (Agilent), a microarray was performed using Agilent mouse whole genome 8 × 60 K (Agilent). Following hybridization to gene arrays, the labeled cDNA was washed and scanned using Agilent Microarray scanner G2505C (Agilent). For detection of significant differences of gene expressions between the THX and PNX groups, each slide image was processed by Agilent Feature Extraction software (version 11.0.1.1).

### Protein extraction and Western blot analysis

Protein expression was evaluated by Western blot analysis. After perfusion with saline, the harvested right superior lobe was homogenized with a denaturing RIPA lysis buffer (Sigma, Stockholm, Sweden) on ice for 15 min. Then, the lysate was centrifuged at 14,000 rpm for 15 min at 4 degrees Celsius, and the supernatants were collected with Laemmli Sample buffer. Sodium dodecyl sulfate-polyacrylamide gel electrophoresis was applied to the supernatants under reducing conditions followed by transfer to a polyvinylidene difluoride membrane for 90 min at 90 V using HorizBlot (Atto, Tokyo, Japan). After blocking nonspecific reactions with Block Ace (Dainippon Pharmaceutical, Osaka, Japan), the primary antibodies for NF-κB p65 (1:1000, C19: Santa Cruz Biotechnology, Dallas, TX) or beta-actin (1:2000, Abcam; Cambridge, UK) were incubated with the blot overnight at 4 degrees Celsius. The secondary antibody, ECL anti-rabbit IgG horseradish peroxidase conjugated antibody (GE Healthcare, UK), was incubated with the blot for 1 h at room temperature. Bands were detected by enhanced chemiluminescnence using ECL Western Blotting Detection Reagents (Amersham Bioscience, Buckinghamshire, UK). Band densitometry was quantified using Image J (U. S. National Institutes of Health, Bethesda, MD). The values were normalized to beta-actin.

### Immunohistochemistry

For immunohistochemistry, the remnant right lung was resected at 12 h or 48 h in both the PNX and THX groups and was inflated with intratracheal instillation 10% buffered formalin at a pressure of 20 cm H_2_O after saline perfusion. The trachea was tied under the pressure, and the lung was fixed in the chest cavity for 48 h. The formalin fixed lung was embedded in paraffin, and cut sagittally in 4 μm sections for hematoxylin and eosin staining and immunohistochemistry. The primary antibodies used were: anti-NF-κB p65 rabbit monoclonal antibody (1:750, ab16502; Abcam) and anti-prosurfactant proteinC (pro-SPC) goat monoclonal antibody (1:1000, C-19; Santa Cruz). The corresponding secondary antibodies (Impress; Vector Laboratories, Burlingame, CA) to the primary antibodies were used. Then they were visualized with 3,3′-diaminobenzidine tetrahydrochloride (Sigma-Aldrich, St. Rouis, MO). One section was selected per animal for each group, and five fields were randomly selected per section. The slides were coded and masked for identity. Positive cells for the each marker were evaluated by YT and HD. Any discrepancies between the observers were resolved with consensus. Nuclei staining positive for NF-κB p65 were counted and expressed as a proportion to total number of epithelial cell nuclei in high power (× 20) field.

### Reagent administration

At 15 min post-surgery, 100 μL of NF-κB inhibitor SN50 (0.1 μmol/ kg; Wako Pure Chemical Industries, Ltd., Osaka, Japan) [20, 21] or vehicle (100 μL of normal saline) was slowly instilled into the nasal cavity as previously described [4, 22].

### Lung dry weight measurement

To evaluate the degree of compensatory lung growth, the lung dry weight 7 days post-surgery was measured after complete drying in a vacuum oven at 95 degrees Celsius, −270 cm H_2_O for 48 h. It was represented as lung dry weight index (LDWI), a ratio of the dry lung weight to body weight [3, 4].

### Measurement of mean linear intercept

The mean linear intercept was measured as previously described [8, 22, 23]. Briefly, 10 equally distributed horizontal lines and 11 evenly distributed vertical lines were drawn over a hematoxylin and eosin staining section. For each line, intercepts with the alveolar wall were counted under light microscopy at 100× magnification. The mean linear intercept was calculated for each mouse as the average ratio of line length divided by the number of alveolar wall intercepts.

### Statistical analysis

All data were represented as a mean ± standard deviation. The unpaired *t* test was performed for compare the difference between two groups. Multiple comparisons were performed using one-way ANOVA with post hoc Tukey test. All statistical tests were two-sided, and *p* values less than 0.05 were considered statistically significant. SPSS software (version 24; SPSS Inc., Chicago, IL) was used for statistical analyses.

## Results

### Gene expression profile in the early phase of pneumonectomy

First, gene microarray analysis was performed to identify significant changes of gene expression in a time-dependent manner. Based on our previous data as described above [3], molecular triggers should be upregulated between 1 h after pneumonectomy when the dilatation of alveolar duct peaks and 24 h after pneumonectomy when alveolar septal cells begin to proliferate. Hence, we focused on a time point at 12 h after pneumonectomy to detect possible molecular triggers for microarray analysis.

**Fig. 1:**
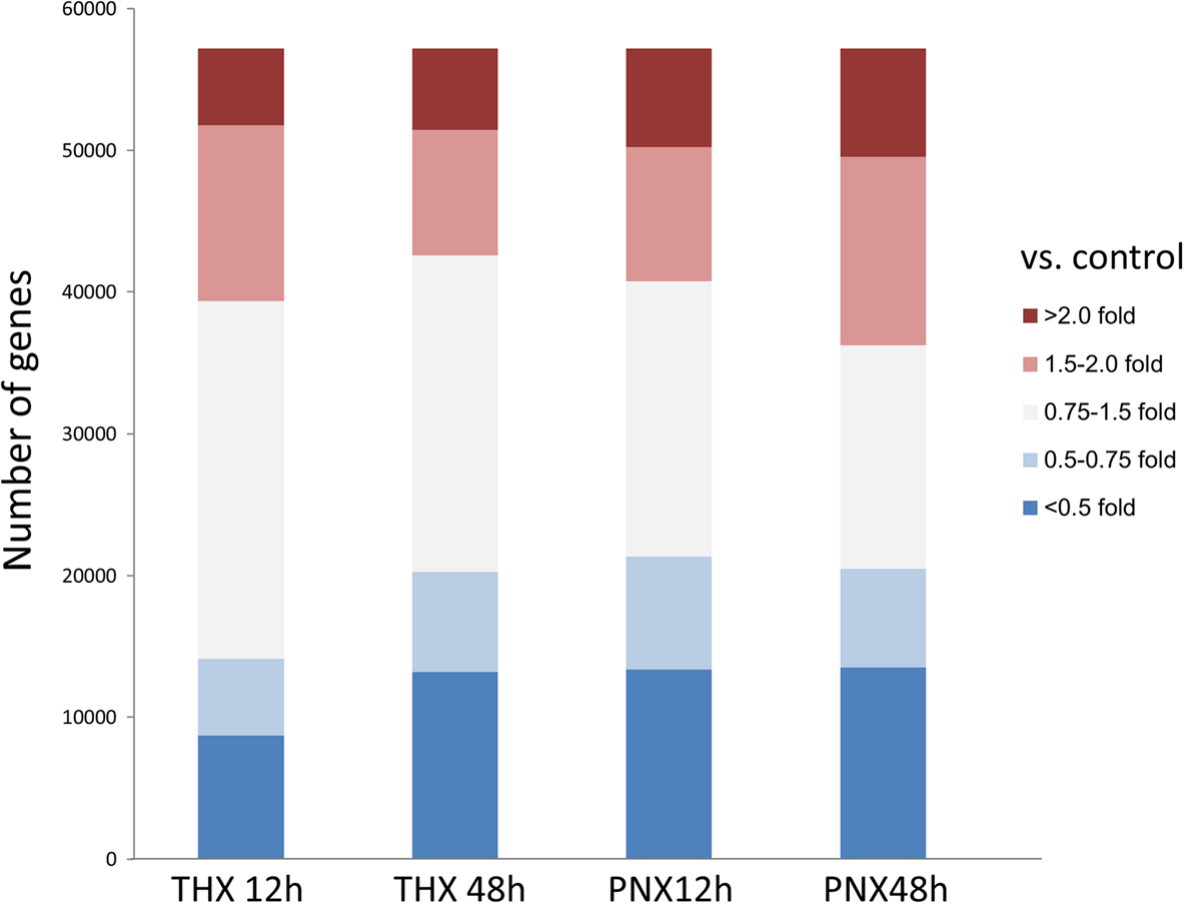
Data summary of global gene expression profiling. Data summary of global gene expression analysis was represented as fold changes to the Control group. Y-axis shows number of genes and bar color representing each fold changes classified into five groups (>2.0 fold, 1.5–2.0 fold, 0.75–1.5 fold, 0.5–0.75 fold, and <0.5 fold)

As shown in Fig. 1, comprehensive gene expression profiling data normalized by the control group revealed that there were 6943 genes expressed greater than 2 fold in the PNX 12 h group. Among these, we selected genes from the 12 h and 48 h THX groups or the 48 h PNX group whose expression level was comparable to the control group. Subsequently, 11 genes which demonstrate relevant time-dependent changes of gene expressions were identified as shown in Table 1. NF-κB could be considered a key factor in CLG because of its gene function library characteristics and literature suggesting its key role in tissue regeneration and cell differentiation [14–16]. The NF-κB gene expression fold changes to the Control group were 3.818-fold in the PNX 12 h group, 1.458-fold in the PNX 48 h group, 1.329-fold in the THX 12 h group, and 1.195-fold in the THX 48 h group.

**Table 1.** Eleven genes which reveal the specific time-dependent changes identified by gene expression microarray

Ref SeqRNA	Gene symbol	Gene name	Fold change at 12 h after pneumonectomy
NM_009702	Aqr	aquarius	2.092
NM_009755	Bmp1	Bone morphogenetic protein 1	2.175
NM_028027	D10Ertd610e	DNA segment, Chr 10, ERATO Doi 610, expressed	2.746
NM_028765	Acoxl	acyl-Coenzyme A oxidase-like	2.309
NM_001017966 /// NM_023544	Ddi2 /// Rsc1a1	DNA-damage inducible protein 2 /// regulatory solute carrier protein, family 1, member 1	2.080
NM_025773	Ube2w	Ubiquitin-conjugating enzyme E2W	2.212
NM_008689	NF-kappa B 1	nuclear factor kappa B subunit 1	3.818
NM_001039652	Oprm1	opioid receptor, mu 1	5.381
NM_021493	A330041B18Rik // Arhgap23	RIKEN cDNA 4933428G20 gene	2.030
NM_018760	Slc4a4	solute carrier family 4 (anion exchanger), member 4	2.095
NM_026417	Yipf4	Yip1 domain family, member 4	2.360

### Time-dependent increase of NF-κB p65 protein levels

**Fig. 2:**
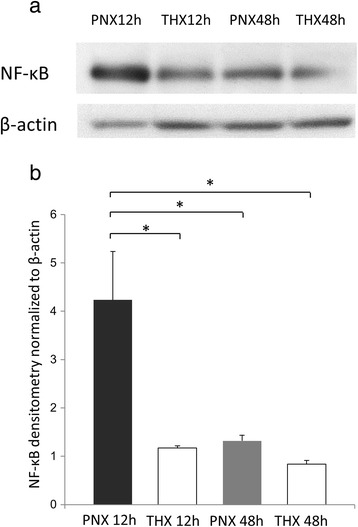
Western blot analysis of NF-κB. **a**. Representative Western blot analysis of the remnant right lung in the PNX 12 h, PNX 48 h, THX 12 h, and THX 48 h groups. **b**. Densitometry of NF-κB was normalized to β-actin (*n* = 4 for each group). Statistical differences were tested using ANOVA with post hoc Tukey test. *p < 0.05, ** *p* < 0.01 between the indicated groups

To identify NF-κB p65 protein expression levels in the remnant right lung, Western blot was performed (*n* = 5 for each group). As shown in Fig. 2a and b, the expression of NF-κB p65 normalized to beta-actin in the PNX 12 h, THX 12 h, PNX 48 h, and THX 48 h groups were 4.23 ± 1.00, 1.18 ± 1.11, 1.32 ± 0.11, 0.84 ± 0.07, respectively. The PNX 12 h group demonstrated significantly higher expression of NF-κB p65 compared to the other three groups (*p* = 0.029 for each). The changes of NF-κB p65 expressions shown by Western blot analysis were consistent with the results of the gene microarray analysis, which showed a transient but significant increase in NF-κB expression at 12 h post-pneumonectomy.

### NF-κB protein expression increases promptly in type 2 alveolar epithelial cells of the right remnant lung after left pneumonectomy

**Fig. 3:**
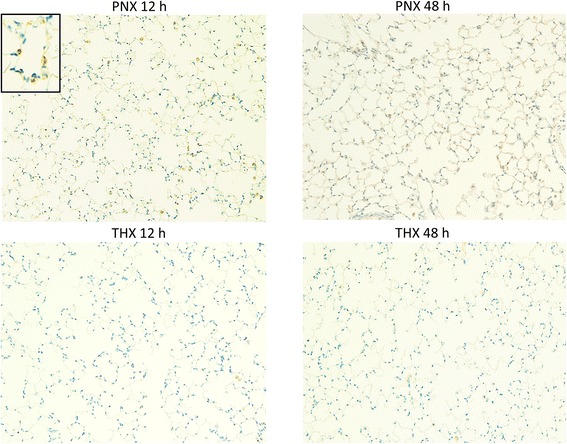
Immunohistochemistry for NF-κB. Representative findings of immunohistochemistry for NF-κB of the remnant right lung (×100 magnification) in the PNX 12 h, PNX 48 h, THX 12 h, and THX 48 h groups. NF-κB positive cells were mainly shown in alveolar epithelium in the PNX 12 h group (inset: higher magnification)

**Fig. 4:**
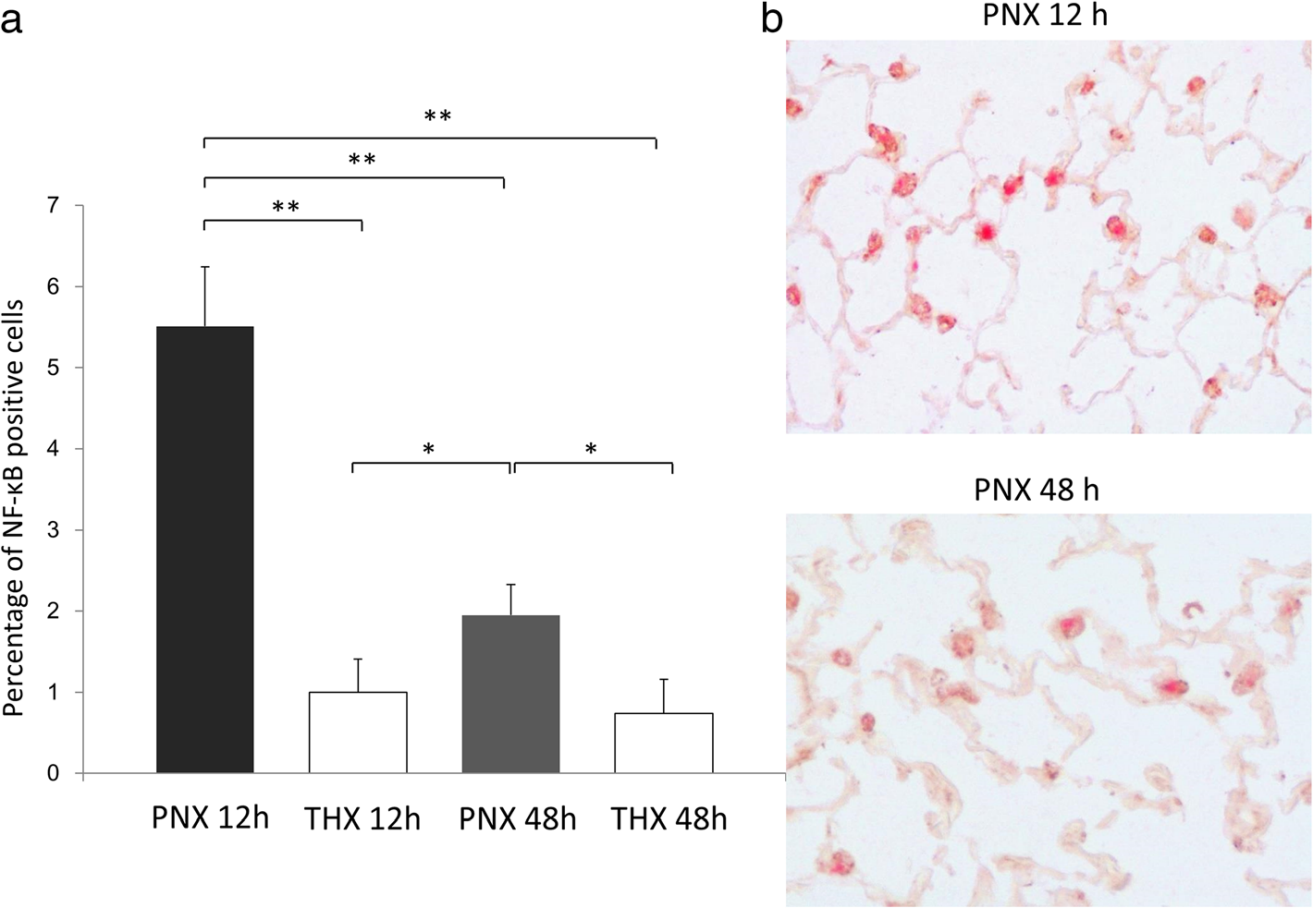
Summary of immunohistochemistry of NF-κB. **a**. NF-κB p65 expression in remnant right lung in each group. Statistical differences were tested using ANOVA with post hoc Tukey test. *p < 0.05, ** *p* < 0.01 between the indicated groups. **b**. Upon double staining for NF-κB and pro-SPC, majority of NF-κB positive cells revealed colocalization with pro-SPC positive immunoreactivity in the remnant right lung at 12 h after pneumonectomy. Double positive cells were fewer at 48 h after pneumonectomy

Upon immunohistochemistry, NF-κB expression was quantified (*n* = 5 for each group). Figure 3 revealed high expression of NF-κB in the nuclei of alveolar epithelial cells in the PNX 12 h group. On the other hand, positive cells were scant in the PNX 48 h group and very few positive cells were present in both the THX 12 h and THX 48 h groups. The proportion of NF-κB positive cells to all epithelial cells was 5.51 ± 0.73% in the PNX 12 h group, 1.00 ± 0.41% in the THX 12 h group, 1.95 ± 0.38% in the PNX 48 h group, and 0.74 ± 0.42% in the THX 48 h group. The percentage of NF-κB positive cells in the PNX 12 h group was significantly higher than those in the other three groups (*p* < 0.001 for each group; Fig. 4a). The NF-κB positive cells in the PNX 48 h group were greater than either the THX 12 h or THX 48 h group (*p* = 0.023 for each). In addition, it should be noted that the inflammatory cell infiltration was not significant in any of the experimental groups.

Furthermore, double staining for NF-κB and pro-SPC in the PNX 12 h and PNX 48 h group was performed to investigate which types of cells expressed NF-κB. As shown in Fig. 4b, colocalization of NF-κB and pro-SPC was shown, suggesting that NF-κB was mainly expressed in type 2 alveolar epithelial cells. The percentage of double positive cells to pro-SPC positive cells of PNX12 hour group was significantly higher than that of PNX 48 h group (27.54 ± 5.89% vs. 8.47 ± 2.02%, *p* < 0.001; Fig. 4b).

### SN50 inhibited compensatory lung growth via decreasing NF-κB

**Fig. 5:**
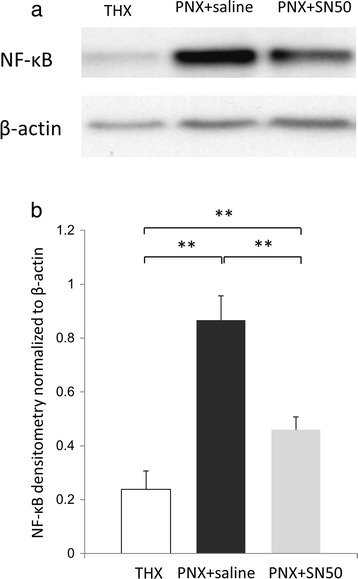
NF-κB inhibition by SN50 administration. **a** Representative Western blot analysis at 12 h after surgery in the THX, PNX + saline, and PNX + SN50 groups. **b** Densitometry of the NF-κB band was normalized to that of β-actin (*n* = 4). Statistical differences were tested using ANOVA with post hoc Tukey test. *p < 0.05, ** *p* < 0.01 between the indicated groups

SN50, a cell permeable peptide inhibiting NF-κB translocation [20, 21], was administered to clarify a influence of NF-κB in this model (*n* = 7 for each group). As shown in Western blot analysis, the NF-κB expression was decreased by SN50 administration at 12 h after pneumonectomy (Fig. 5a). The densitometry value of NF-κB expression normalized to β-actin was 0.238 ± 0.068 in the THX group, 0.866 ± 0.091 in the PNX + saline group, and 0.459 ± 0.048 in the PNX + SN50 group. The NF-κB expression was significantly decreased in the PNX + SN50 group compared to the PNX + saline group (*p* < 0.001; Fig. 5b), but was significantly higher than the THX group (*p* = 0.004). Also, there is significant difference in the NF-κB expression between the PNX + saline group and the THX group (*p* < 0.001). Taken together, the expression of NF-κB was not completely, but significantly, decreased by SN50 administration following pneumonectomy.

**Fig. 6:**
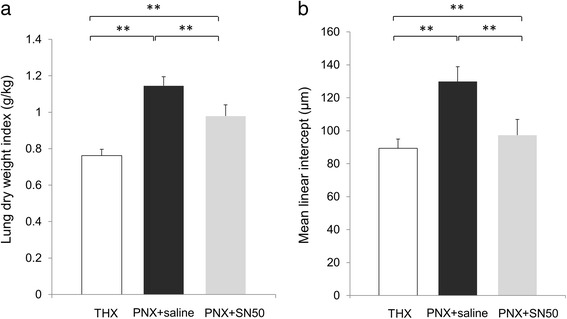
Changes in lung dry weight index with SN50 administration. **a**. Lung dry weight index of the remnant right lung at 7 days after surgery in the THX, PNX + saline, and PNX + SN50 groups (*n* = 7 for each group). B).Mean linear intercept of the remnant right lung at 7 days after surgery in the THX, PNX + saline, and PNX + SN50 groups (*n* = 7 for each group). Statistical differences were tested using ANOVA with post hoc Tukey test. *p < 0.05, ** *p* < 0.01 between the indicated groups

We then measured LDWI of the right lung in the three groups to compare the degree of lung growth between these treatment groups (*n* = 7 for each group). At 7 days after treatment, the LDWI was 0.763 ± 0.034 g/kg in the THX group, 1.145 ± 0.049 g/kg in the PNX + saline group, and 0.979 ± 0.062 g/kg in the PNX + SN50 group. SN50 administration following pneumonectomy induced significantly decreased LDWI compared to the saline administration following pneumonectomy (*p* = 0.004; Fig. 6a). The LDWI in both the PNX + saline and the PNX + SN50 groups were significantly greater than that in the THX group (*p* < 0.001 for each). In addition, the mean linear intercept (*n* = 7 for each group) was 89.29 ± 5.71 μm in the THX group, 129.86 ± 8.99 μm in the PNX + saline group, and 97.29 ± 9.57 μm in the PNX + SN50 group. The mean linear intercept of the PNX + SN50 group was significantly smaller than that of the PNX + saline group (*p* = 0.002; Fig. 6b). Inhibition of NF-κB by SN50 administration induced suppression of CLG at 7 days after pneumonectomy.

## Discussion

The most significant finding of the current study is that NF-κB inhibition by SN50 administration significantly suppressed CLG as shown in the results of LDWI and mean linear intercept. In addition, the NF-κB gene expression transiently but significantly increased, which was consistent with Western Blot and immunohistochemistry analyses that showed similar time-dependent changes, mainly in type 2 alveolar epithelial cells. To our knowledge, this is the first report demonstrating a role of NF-κB in CLG following pneumonectomy.

CLG has reemerged as a translatable lung regeneration model in recent years, which has been investigated since 1970s [24, 25]. Our prior studies demonstrated that the morphology of the alveolar area drastically changed during CLG [3]. At 1 h after pneumonectomy, remnant right lung showed significantly enlarged alveolar ducts and at 24 h after pneumonectomy, the Ki-67 expression of alveolar septal cells gradually increased, mainly contributing to increased number of alveoli. In addition, the morphological changes in CLG resembled septation in normal lung development mediated by TTF-1, a key regulator in the early phase of CLG [3, 4]. Due to the time between the observation of enlarged alveolar ducts at one hour and increased proliferation of alveolar septal cells at 24 h, we focused on a 12 h time point after pneumonectomy to investigate a possible molecular target in the very early phase of CLG. In the current study, the NF-κB protein expression was transiently but significantly increased at 12 h after pneumonectomy prior to decreasing to non-significant levels at 48 h after pneumonectomy. Of note, the NF-κB was mainly expressed in type 2 alveolar epithelial cells similar to the type 2 alveolar epithelial cell expression of TTF-1 in our previous study [3]. These data may support NF-κB in type 2 epithelial cells as a key mediator in CLG following left pneumonectomy.

Interestingly, the current data suggest that the NF-κB plays a critical role in not only CLG but also in liver regeneration after partial hepatectomy. A time point when NF-κB expression peaked was reportedly 12 h after resection in liver regeneration model which is consistent with previous literature [26]. Although the detailed anatomy and cellular architectures are different, the development of the two organs has similarity in that two different vascular systems and functional ducts form a basis of lobular structures. In addition, in a recent report of Bi-Sen, angiocrine factors are largely involved in the process of CLG [5], mirroring their contribution to liver regeneration [16]. Moreover, the vascular endothelial growth factor signaling pathway can be upregulated by NF-κB signaling, which contributes to maintaining regenerative capacity of bone marrow mesenchymal stem cells [27, 28]. Given these factors have important roles in the tissue regeneration of various organs; our findings may have impact beyond clarifying the molecular trigger of CLG.

There are two previous publications investigating global gene expression profiling after pneumonectomy to date [8, 9]. Wada et al. showed that NF-κB inhibitor zeta gene expressions, which may change in parallel with NF-κB itself, increased in time points up to 3 months (except at 6 months) post-pneumonectomy in a rat model (3 days, 7 days, 1 month, and 3 months) [8], with the highest level of expression found at day 3 post-pneumonectomy. Although there seems a difference in time-dependent expression patterns, which could be explained by difference of animal species that were used, it may support the relevance of NF-κB signaling to CLG. In another report, NF-κB expression was down-regulated at 6 h after pneumonectomy [9]. Since NF-κB was shown to up-regulated by surgical stress including thoracotomy as well as pulmonary resection [29], we used a negative control group without any surgical procedure other than sedation and intubation. However, Paxson et al. set sham operation group (THX group) as a negative control, which can potentially increase NF-κB expression level. This may be one of the possible reasons for the different result from ours. Moreover, neither of the studies confirmed the changes of NF-κB gene expression by PCR or the NF-κB protein expression levels.

Mechanical stretch of epithelial cells due to the rapidly increased intrathoracic negative pressure right after pneumonectomy was considered as one of the possible triggers in CLG [3, 30]. Sebag and colleagues demonstrated that mechanical stretch of lung epithelial cells caused increased expression of NF-κB in vitro [31]. Also, overinflation of the remnant lung following left pneumonectomy may cause increased secretion of proinflammatory cytokines that are associated with NF-κB in mice [32]. In addition evidence of pro-inflammatory cytokines and NF-κB in a remnant lung, Ogawa et al. demonstrated a similar NF-κB and proinflammatory cytokine relationship in a ventilator-induced lung injury model [33]. Therefore, it is reasonable that NF-κB was upregulated by mechanical stretch of epithelial cells in the remnant right lung after pneumonectomy, which is consistent with morphological findings in our previous study [3]. On the other hand, there may be a criticism that the main cause of the increased NF-κB expression after pneumonectomy can be explained by infiltration of inflammatory cells in the remnant lung [34, 35]. Contrary evidence to this criticism include findings that the NF-κB expressions were mainly localized to type 2 alveolar epithelial cells on immunohistochemistry and that inflammation was not histologically relevant in either the PNX 12 h group or the THX 12 h group. Importantly, type 2 alveolar epithelial cells have the potential to maintain structure and function of alveolar epithelial cells [36]. These points highlight the importance of NF-κB expression that was mainly shown in type 2 alveolar epithelial cells. However, a possibility remains that minor infiltration of inflammatory cells can contribute to the increased NF-κB levels observed in CLG after pneumonectomy. Altogether, the elevated expression of NF-κB in the very early phase of CLG may be associated with molecular mechanism. Moreover, it might be a key mediator between mechanical stretch and downstream molecular cascades.

Administration of SN50, an inhibitor of NF-κB translocation [18, 19], suppressed NF-κB expression at 12 h after pneumonectomy. Wissel et al. reported that the nuclear translocation inhibitor, SN 50, significantly decreased nuclear NF-κB p65, but did not change the cytoplasmic NF-κB p65 level in type 2 epithelial cells [37]. This may support our data, even though we did not look at the exact translocation of NF-κB. Moreover, down-regulation of NF-κB p65 by SN50 administration in vivo was consistent with a report of Sun et al. [38]. The down-regulated NF-κB protein expression caused significant suppression of CLG that was represented as the LDWI and mean linear intercept at 7 days after pneumonectomy. The lower mean linear intercept means small alveolar space including alveoli and alveolar ducts. The current finding may suggest that SN50 disturbed alveolar enlargement after pneumonectomy that is considered to be the possible mechanical trigger in the early phase postpneumonectomy [3, 39]. On the other hand, previous study (reference 8) demonstrated that increased mean linear intercept kept higher level that is consistent with our findings. The increased mean linear intercept in the PNX group could be a persistent alveolar enlargement to be decreasing to the similar level to the PNX + SN50 group by further alveolar growth thereafter, even though sequential mechanisms and the time-course are still unknown. NF-κB signaling is controlled by canonical and/or non-canonical pathways. There are several ways blocking NF-κB such as IκBα activation [40], reducing deoxyribonucleic acid binding activity to NF-κB [41], and inhibiting translocation of NF-κB [42]. Among these, translocation of NF-κB can be the most important functional mechanism as previously described [42]. In this study, SN50 demonstrated suppression of NF-κB in our model of CLG following left pneumonectomy, thereby suggesting a crucial role of NF-κB in this model. On the other hand, NF-κB inhibition by SN50 administration caused some degree of CLG that is consistent with similar degree of the increased NF-κB expression in the PNX + SN50 group compared with the THX group. One limitation of utilizing SN50 is that it can affect many proinflammatory cytokines and molecules that might have influence on the CLG process. Also, we have not investigated the downstream signaling pathway of NF-κB in CLG at this point. Further analysis is required to clarify the molecular mechanisms of CLG which might have potential to facilitate functional and volumetric recovery after lung resection in adult humans.

## Conclusions

We have demonstrated that the NF-κB expression was transiently but significantly increased in the early phase of CLG. Inhibition of NF-κB expression by SN50 administration significantly suppressed CLG. This suggests that NF-κB may have a crucial role in compensatory lung growth. This study identified molecular triggers that may also be clinically useful regulators of compensatory lung growth.

## Abbreviations

CLG: Compensatory lung growth
LDWI: Lung dry weight index
NF-κB: That nuclear factor-kappa B
pro-SPC: Anti-prosurfactant proteinC
TTF-1: Thyroid transcription factor 1
